# Unusual Presentation of Cerebral Fat Embolism Syndrome Post-femur Fracture: A Case Study and Diagnostic Insights

**DOI:** 10.7759/cureus.64819

**Published:** 2024-07-18

**Authors:** Rahul Salunkhe, Dattatray Bhakare, Rishabh Aggarwal, Sarthak Walia

**Affiliations:** 1 Orthopaedics, Dr. D. Y. Patil Medical College, Hospital and Research Centre, Dr. D. Y. Patil Vidyapeeth, Pune, IND

**Keywords:** management, diagnosis, neuroimaging, atypical presentation, femur shaft fracture, cerebral fat embolism syndrome

## Abstract

Cerebral fat embolism syndrome (CFES) is a rare but certainly devastating impediment following long bone fractures. The diagnosis of CFES primarily depends on identifying clinical manifestations like respiratory distress, petechial rash, and neurological symptoms. However, in rare instances, CFES can manifest with atypical or absent clinical features, posing diagnostic challenges.

Here, we present a rare case report of a woman in her 20s who developed CFES after suffering a femur shaft fracture devoid of conventional clinical features. The diagnosis of CFES was built upon clinical suspicion and a typical MRI brain finding of a *starfield pattern*.

Our case highlights the importance of including CFES in the differential diagnosis of neurological deterioration, especially after long bone fractures. We suggest early plate osteosynthesis to stop more emboli from forming in people with FES, as well as continuous neuromonitoring and a reminder that CFES can show up without any other signs or symptoms in the body.

## Introduction

The occurrence of fat droplets in the bloodstream causes the uncommon condition known as fat embolism syndrome (FES), which frequently develops following long bone fractures or intramedullary nailing procedures. Symptoms of FES typically develop within the first 24 to 72 hours after the event [1]. The classic signs of FES encompass respiratory distress, petechial rash, and neurological symptoms. Nevertheless, the presentation of FES can vary significantly, and at times, the classical clinical features may not be present, posing challenges in diagnosis.

The clinical criteria for cerebral fat embolism (CFE) are not always very specific, although this is the main method of diagnosis. Therefore, MRI is the gold standard for diagnosis, especially diffusion-weighted imaging (DWI) and susceptibility-weighted imaging (SWI) sequences. These MRI techniques are excellent at detecting blood products, which is crucial for identifying CFE [2].

Herein, we present an unusual case of cerebral FES secondary to a femur fracture that developed within 24 hours of trauma and exhibited profound neurological impairment without any other systemic findings.

## Case presentation

A female in her 20s was brought to the emergency department with a reported history of a motor vehicle accident (MVA) and no history of loss of consciousness. Upon admission, the patient was drowsy, had stable vital signs, and scored E4V5M6 on the Glasgow coma scale (GCS). Pupils were round and bilaterally cognizant of light.

**Figure 1:**
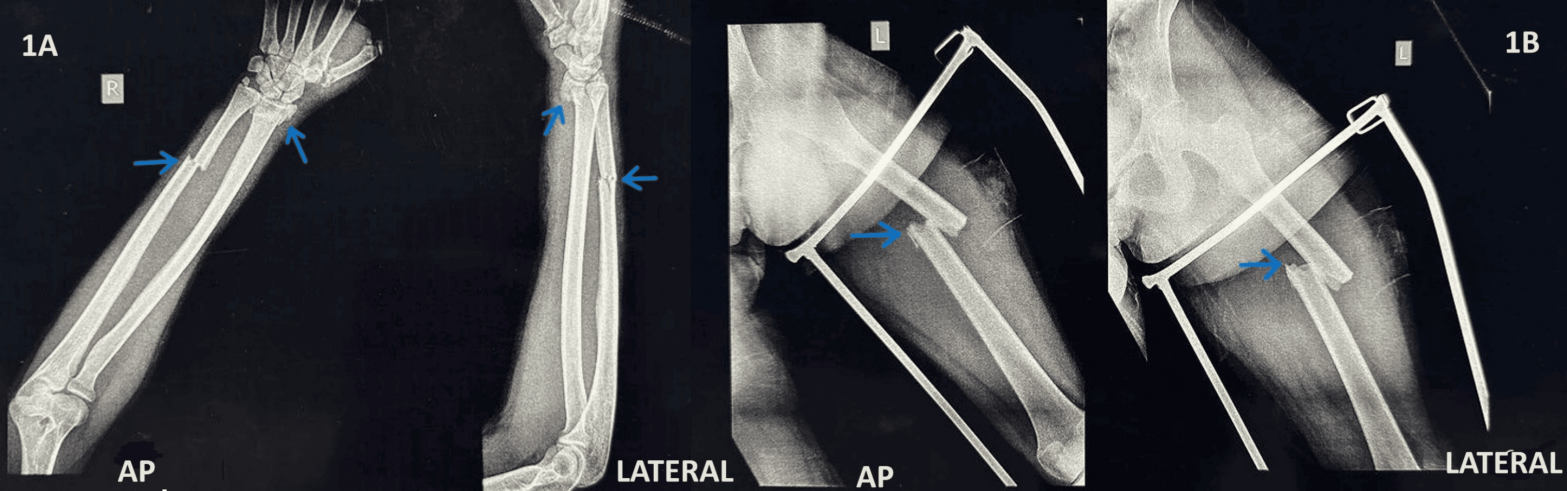
Preoperative plain radiograph. (A) Arrows showing right distal end radius fracture and distal one-third ulna shaft fracture in anteroposterior (AP) and lateral views. (B) Arrows showing left femur shaft fracture in AP and lateral views.

On physical examination, the patient had swelling, tenderness, and crepitus in the left thigh, right wrist, and right forearm. The left femur shaft fracture, the right distal end radius fracture, and the right ulna shaft fracture were identified on a plain radiograph, as represented in Figure 1. Vascularity in both the upper and lower extremities was intact. The patient had a posttraumatic bilateral pleural effusion. Initial stabilization of the fractures was done and was started on antibiotics.

**Figure 2:**
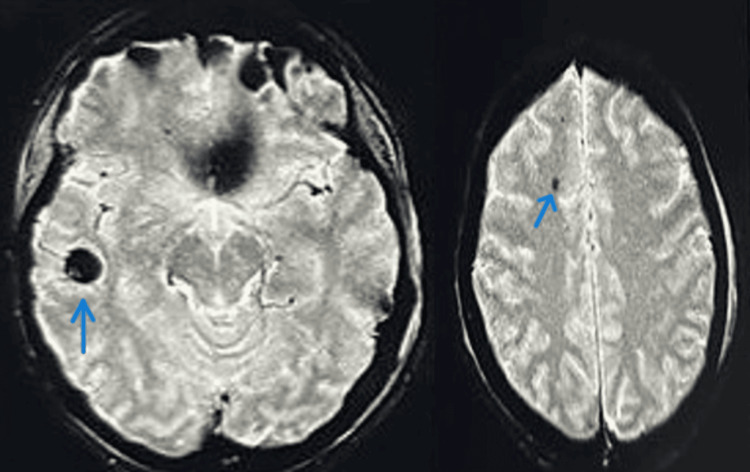
MRI brain: arrows showing hypodense lesions in the cerebral hemisphere due to fat embolism.

A sequential head CT was done, which was suggestive of right temporoparietal contusions with multiple small hemorrhages. Lab investigations were normal. The patient's femur fracture was prioritized for early fixation, and surgery was set for the same day. However, during preoperative evaluation, the patient was found to have altered mental status, slurred speech, confusion, and had an episode of generalized tonic-clonic seizure, and GCS dropped to 8 within 24 hours of trauma. She was emergently sedated and intubated. Brain MRIs taken at a later date confirmed the presence of fat emboli. Figure 2 shows widespread hypodensity on both sides of the brain, but no evidence of mass impact or midline shift was detected. An echocardiogram revealed normal ventricular function without any signs of a blood clot. CT pulmonary angiography (CTPA) was done, which showed signs of pulmonary artery dilation suggestive of pulmonary artery hypertension, but there were no signs of pulmonary thromboembolism. The patient was given an injection of methylprednisolone and low-molecular-weight heparin right away. She was also given hypertonic saline, antiepileptic drugs, and a mix of mannitol and glycerin, and the condition was constantly monitored while she was sedated and intubated. The bilateral lower limb color Doppler was normal. A dilated fundus examination was performed to look for retinal evidence of fat emboli; however, this was not found. Ophthalmology was consulted in this regard. Moreover, there were no signs of petechial rash.

**Figure 3:**
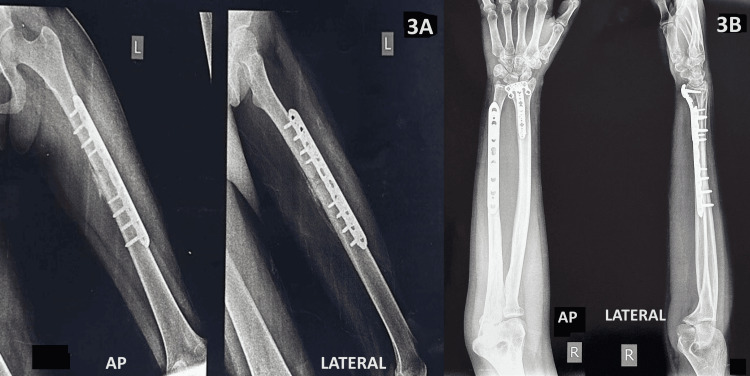
Postoperative plain radiograph. (A) Open reduction internal fixation with plating for femur shaft fracture in anteroposterior (AP) and lateral views. (B) Open reduction internal fixation with plating for ulna shaft fracture and distal end radius fracture in AP and lateral views.

As soon as it was safe to do so in an emergency, the patient was taken to get their fractures fixed. She had open reduction internal fixation with plating for a femur shaft fracture, an ulna shaft fracture, and a distal end of a radius fracture, as shown in Figure 3.

Over the subsequent days, the patient's neurological status gradually improved with the resolution of the confusion and weakness. Follow-up imaging demonstrated regression of the cerebral lesions. The patient was eventually weaned off mechanical ventilation and discharged home with an orthopedic follow-up.

## Discussion

Although FES is rare, nearly all patients with multisystem trauma have asymptomatic fat embolism. The mortality rate for long bone fracture sequelae such as FES and CFE ranges from 5% to 15%. Respiratory failure makes up the majority of mortality. The literature reports an incidence of 0.9% to 2.2% for CFE [3] and an incidence of 1% to 10% for FES [1]. 

Two main theories are used to explain the precise pathophysiology of CFE.

Mechanical theory

According to mechanical theory, when the bone marrow has an injury, it releases little fat particles, ranging from 7 to 10 µm in size, from its fat cells. They make their way into the veins after that. If an intrapulmonary shunt or patent foramen ovale (PFO) is present, they can go via systemic circulation. Because of the risk of a systemic embolism in the left circulation, breathing may become difficult. Even though it is less prevalent, fat emboli that are larger than 20 µm can nevertheless pass through the pulmonary filter when a PFO is not present [4].

Chemical theory

According to this chemical theory, several tissues undergo lipolysis when plasma mediators are released during the acute phase of trauma. As a result, the end product is fatty acids that cause a cascade of events that begins with a buildup of inflammation and ends with the gradual destruction of tissues due to vessel obstruction. Clotting occurs as a result of platelet clumping, endothelial injury, and bleeding. The delayed symptoms observed in CFE are due in part to all of these factors [5].

Both hypotheses concur that fat embolism leads to typical microinfarctions, vasogenic edema, and pinpoint hemorrhages in the brain.

Neurological signs are apparent in about 80% to 85% of cases of FES [1]. These typically manifest as sudden brain dysfunction with vague symptoms or initial changes in mental state that can rapidly progress from confusion to unconsciousness. Additional neurological symptoms may involve specific signs like one-sided paralysis, speech difficulties, inability to perform learned movements, failure to recognize objects, seizures, or abnormal muscle contractions. Autonomic nervous system dysfunctions, including rapid heartbeat, profuse sweating, and elevated body temperature, are also common, especially when the basal ganglia are affected.

One of the three main diagnostic criteria listed by Gurd and Wilson for FES is neurological symptoms. The other two criteria include respiratory involvement, which often manifests earliest in approximately 75% of cases, and the presence of a petechial cutaneous rash. The rash is commonly thought to be pathognomonic for FES, but it is absent in 50% to 80% of cases and usually appears in the upper third of the chest, the axillae, and the conjunctiva.

In our instance, the patient only showed neurological involvement and did not exhibit any respiratory distress or petechial rash. This highlights the variability in the clinical presentation of FES and underscores the importance of considering FES in the differential diagnosis even when some classical signs are absent.

A black backdrop with numerous tiny patches of increased signal intensity is a common occurrence in DWI. This gives rise to the easily identifiable *starfield pattern* as early as four hours following the onset of brain damage symptoms. The diagnosis of CFE primarily relies on clinical evaluation, although specific brain MRI findings can significantly bolster the diagnosis. A black backdrop with numerous tiny patches of increased signal intensity is a common occurrence in DWI. This gives rise to the easily identifiable *starfield pattern* as early as four hours following the onset of brain damage symptoms. The damage caused by CFE is mostly found in the cerebellar hemispheres, the corpus callosum, the basal ganglia, the brainstem, and the subcortical and periventricular white matter [2].

Microhemorrhages can be found on T2*-weighted imaging (T2WI) sequences between seven days and one month after the injury in areas with less signal intensity. Susceptibility-weighted imaging (SWI) surpasses T2WI in sensitivity for detecting pinpoint hemorrhages. In SWI, there are small areas where the signal intensity is lower, and the lesions are spread out over a much larger area than in other sequences, such as DWI. This heightened sensitivity renders SWI especially useful in diagnosing CFE and gauging the degree of hemorrhagic damage [6].

The main differential diagnosis for CFE in the context of traumatic injury is diffuse axonal injury (DAI). However, a key difference is that neurological symptoms in DAI typically do not present after a lucid interval, which can be seen in CFE. MRI scans of people with DAI show abnormalities that are hyperintense on both the DWI and T2-FLAIR sequences. These are mostly found at the gray-white matter junction, the corpus callosum, the basal ganglia, the brainstem, and sometimes in the cerebellum. These imaging features can help tell the difference between DAI and CFE. The *starfield pattern* on DWI and the large petechial hemorrhages on SWI are more typical of CFE [2].

Early fracture fixation is a cornerstone in the management of patients with FES; however, definitive surgical fixation must be tailored to the patient's clinical stability. Our patient serves as evidence that the choice of definitive fixation depends on their overall clinical status. To establish the best time and type of fixation, Pape et al. suggest classifying patients as either stable, unstable, borderline, or in extremis, depending on their clinical situation. Stable patients can proceed with definitive fixation, while those who are unstable, borderline, or in extremis may benefit from temporary external fixation until they stabilize [7].

There is a strong correlation between early immobilization, preferably begun within 12 to 24 hours following a traumatic injury, and a marked reduction in the frequency of FES when contrasted with delayed therapy [8]. Additionally, fewer pulmonary problems and shorter hospital stays have been associated with early fixation [8]. However, specialists are still debating which surgical fixing method is best. The algorithm developed by Pape et al. emphasizes the critical need for efficient medical therapy in patients suffering from FES to facilitate timely surgical intervention. There is a clear correlation between promptly casting an injured person, ideally within 12 to 24 hours, and a significant reduction in the occurrence of FES cases when contrasted with therapeutic delays [8]. Additionally, fewer pulmonary problems and shorter hospital stays have been associated with early fixation [8]. However, specialists are still debating which surgical fixing method is best.

A study by Bosse et al. found no significant difference in the occurrence of acute respiratory distress syndrome between intramedullary nailing with reaming and plate fixation [9]. According to Mellor and Soni, blunt reamers, undreamed nails, and intramedullary lavage may cause less damage to bone marrow and may help stop FES from spreading [10]. Conversely, Helttula et al. concluded that reamed versus unreamed nails exhibit no significant difference in their impact on hemodynamics, although they noted that unreamed nails were associated with increased postoperative oxygen consumption [11]. The reamer-irrigator-aspirator also greatly reduced the number of fat emboli seen on a transesophageal echocardiogram [8]. This was shown by Dunn et al. Given these conflicting findings, there is currently no consensus guideline regarding the preferred fixation method, underscoring the necessity for a unified guideline to facilitate real-time decision-making.

## Conclusions

In conclusion, this case report shows how important it is to keep a close eye out for cerebral fat embolism syndrome (CFES) in people who have long bone fractures, even if they do not have any of the classic symptoms. Ruling out clinical suspicions, early recognition, and intervention are paramount in preventing adverse outcomes associated with CFES.

We specifically suggest early plate osteosynthesis to stop further emboli from forming in people with FES, as well as ongoing neuromonitoring, emphasizing that CFES can present without any other signs or symptoms in the body.
